# First application of the distal radial approach for severe mechanical surgical aortic valve paravalvular leak transcatheter closure with a double vascular plug: a case report

**DOI:** 10.1093/ehjcr/ytae366

**Published:** 2024-07-25

**Authors:** Viktor Sasi, Géza Fontos, Árpád Kormányos, Márton Vértesaljai, Zoltán Ruzsa

**Affiliations:** Division of Invasive Cardiology, Department of Internal Medicine, Medical Faculty, Albert Szent-Györgyi Clinical Center, University of Szeged, Semmelweiss str. 8, 6725 Szeged, Hungary; Gottsegen National Cardiovascular Center, Budapest, Hungary; Division of Invasive Cardiology, Department of Internal Medicine, Medical Faculty, Albert Szent-Györgyi Clinical Center, University of Szeged, Semmelweiss str. 8, 6725 Szeged, Hungary; Gottsegen National Cardiovascular Center, Budapest, Hungary; Division of Invasive Cardiology, Department of Internal Medicine, Medical Faculty, Albert Szent-Györgyi Clinical Center, University of Szeged, Semmelweiss str. 8, 6725 Szeged, Hungary

**Keywords:** Case report, Surgical aortic valve replacement, Aortic paravalvular leak, Transcatheter paravalvular leak closure, Distal radial approach, Vascular plug

## Abstract

**Background:**

Severe aortic paravalvular leaks (PVLs) after surgical mechanical aortic valve replacement (AVR) represent a high risk for congestive heart failure, haemolysis, and infective endocarditis. This is the first reported case of distal radial artery (DRA) access for severe mechanical aortic PVL closure with a sequential double vascular plug guided by computed tomography angiography (CTA), transoesophageal echocardiography (TOE), and 3D TOE in an acute setting.

**Case summary:**

A 51-year-old male presented with significant mixed aortic valve disease. Aortic valve replacement was performed (Slimline Bicarbon A-25 mm) according to guidelines. Four and 16 days later, a re-exploration was carried out due to pericardial effusion. Four months after discharge from rehabilitation, the patient was readmitted due to worsening dyspnoea on exertion and then at rest. Transthoracic echocardiography, TOE, and consequently, CTA, revealed severe PVL, following which the procedure of transcatheter PVL closure was chosen, with a preference for DRA access. After a CTA scan analysis and angiographic, TOE, and 3D TOE visualization of the leak, a 14/5 mm and a 10/5 mm vascular plug (AVPIII) were deployed to achieve good results. A 9-month clinical, echocardiographic, and CTA follow-up revealed good long-term results.

**Discussion:**

For transcatheter PVL closure, CTA is helpful for not only vascular access planning, but also a visualization of the magnitude of the leak, location, and device planning. This case report demonstrates that the distal radial approach is feasible in patients with severe mechanical aortic valve PVL retrograde transcatheter closure. DRA access could possibly represent less bleeding and vascular access site complications when compared with femoral access and has some potential advantages over regular radial access.

**Learning points**

- Severe aortic paravalvular leaks (PVLs) after either surgical mechanical/biological aortic valve replacement (AVR) or transcatheter AVR represent a high risk for congestive heart failure, haemolysis, and infective endocarditis and also represent poor prognosis for the patient.
- Transcatheter closure of such leaks delivers significantly less overall risk compared with reoperation.
- Distal radial artery access is feasible for aortic PVL closure and has numerous advantages over femoral and radial access.

## Introduction

Paravalvular leak (PVL) is a defect between the outer skirt of the surgically sutured or transcatheter implanted bioprosthesis and the native valve annulus, resulting in regurgitation of varying severity. The rate of prevalence of aortic PVLs ranges from 6 to 18%, but it can even be as high as 47%,^1,2,3^ as reported in some retrospective studies; however, only a small minority of the detected leaks requires further treatment.

Severe aortic PVLs after surgical mechanical aortic valve replacement (AVR) represent a high risk for congestive heart failure, haemolysis, and infective endocarditis and could indicate poor prognosis for the patient.^3^ This is the first reported case of a distal radial artery (DRA) approach used for severe mechanical aortic PVL closure with a sequential double vascular plug guided by computed tomography angiography (CTA), transoesophageal echocardiography (TOE), and 3D TOE in an acute setting.

## Summary figure

**Table:** 

11/02/2022	Transthoracic echocardiography (TTE): ejection fraction: 43%. No resting regional wall motion abnormality detected. Mixed aortic valve disease verified. Stenosis gradient: 74/47 mmHg. Aortic valve area: 0.33 cm^2^, aortic insufficiency grade: II–III, pressure half time: 222 ms. Tricuspid valve insufficiency grade: I–II with a pulmonary pressure of 70 mmHg Coronary angiography: non-significant circumflex artery lesion
01/03/2022	Surgical aortic valve replacement: LivaNova Bicarbon Slimline A-25 mm mechanical valve
05/03/2022	Reoperation due to major haematoma formation, which caused compression mainly on the right atrium and ventricle. No active bleeding was discovered
17/03/2022	Repeat cardiac surgery to evacuate newly formed pericardial haematoma that compressed the right side of the heart
30/03/2022	Discharge to rehabilitation facility
26/09/2022	Readmission due to progressing dyspnoea. Transoesophageal echocardiography (TOE) and 3D TOE: severe aortic paravalvular leak (PVL) confirmed outside the surgical valve ring, located at the left coronary sinus. Haemolytic anaemia (Hgb: 92–82 g/dL) detected, brain natriuretic peptide level high (7244 pg/mL). Computed tomography angiography confirms high-volume PVL and pulmonary congestion
03/10/2022	Heart team decision: transcatheter PVL closure due to very high surgical mortality risk prediction
05/10/2022	Successful PVL closure performed with two AVPIII plugs, access site: left distal radial artery
13/10/2022	Discharge TTE: good mechanical valve function and clinically not significant residual leak
27/07/2023	Control TOE and 3D TOE confirm good plug positions and mechanical valve function. Residual PVL: remains small, no further worsening during the observation period. Conservative therapy maintained. Vascular ultrasound confirms a patent distal radial artery

## Case presentation

**Figure 1:**
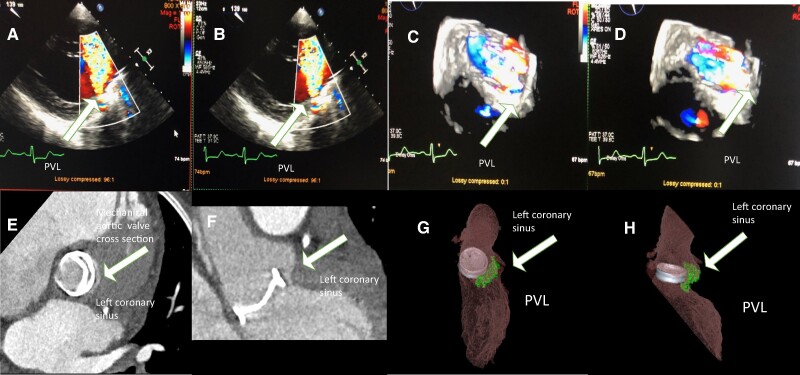
(*A*, *B*) Transoesophageal echocardiography images of a large-sized mechanical aortic valve. A white arrow points at the location in the left coronary sinus. (*C*, *D*) Three-dimensional TOE images of the location of the large-sized mechanical aortic valve paravalvular leak. The white arrows point at the large ostium of the leak. (*E*, *F*) Computed tomography angiography images of the mechanical aortic valve and the detected paravalvular leak. The white arrows point at the leak location. (*G*, *H*) A three-dimensional reconstruction of the mechanical surgical valve, paravalvular leak, and regurgitation volume. The white arrows point at the leak location in the left coronary sinus.

**Figure 2:**
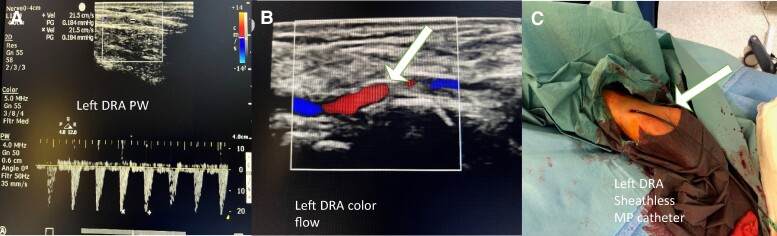
(*A*) An ultrasound image of the planned puncture site of the left distal radial artery (snuffbox). Peak velocity is 21.5 cm/s. (*B*) A colour ultrasound image of the planned puncture site of the left distal radial artery (snuffbox). A white arrow points at the left distal radial artery. (*C*) An image of the left distal radial artery puncture site at the end of the procedure just before taking out the Sheathless guide catheter.

**Figure 3:**
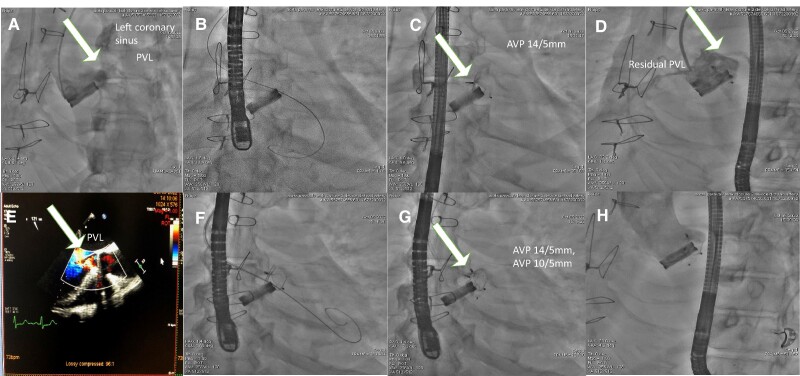
(*A*) Aortography revealing a large-volume paravalvular leak between the outer mechanical valve body and the annulus. A white arrow points at the leak. (*B*) The Confida wire position is carefully checked from different projections to make sure that it is outside the mechanical aortic valve ring. (*C*) A 14/5 mm AVPIII device is released and does not influence valve leaflet motion. A white arrow points at the device. (*D*) Control angiography reveals a decreased but still non-negligible volume paravalvular leak. A white arrow points at the residual leak after AVP 14/5 mm placement. (*E*) Intraoperative transoesophageal echocardiography after the first AVPIII (14/5 mm) release demonstrates still a large-volume leak. (*F*) A careful position check of the Confida wire demonstrates a good position of the wire in between the mechanical valve ring and the prior-implanted AVPIII (14/5 mm). (*G*) The picture after deployment of the second AVPIII (10/5 mm). A white arrow points at the double AVP devices. (*H*) Final angiography demonstrates a small-volume paravalvular leak.

**Figure 4:**
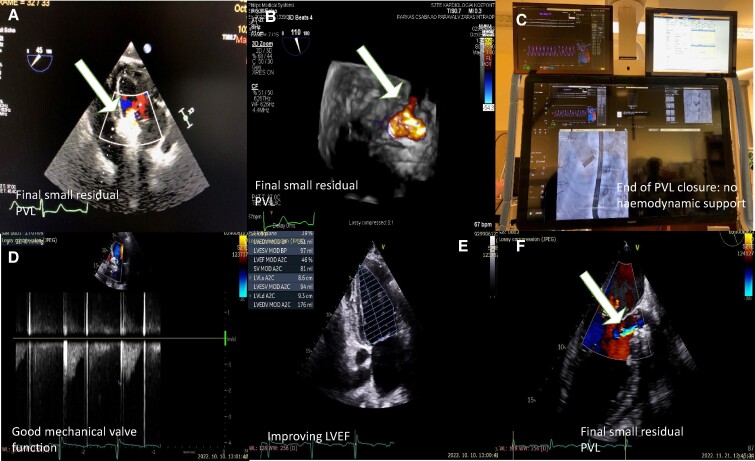
(*A*) Final intraoperative transoesophageal echocardiography demonstrates a good closure result. A white arrow points at the site of plug deployment. (*B*) The final intraoperative three-dimensional transoesophageal echocardiography image demonstrates a good closure result. A white arrow points at the residual small leak. (*C*) Procedure finish, no haemodynamic support. (*D*) Discharge transthoracic echocardiography shows good mechanical valve function. (*E*) Discharge transthoracic echocardiography shows early recovery of left ventricular function. (*F*) Discharge transthoracic echocardiography shows a slight paravalvular regurgitation. A white arrow points at the small residual leak.

A 51-year-old male patient was admitted to the university hospital with chest discomfort, dyspnoea, and decreased exertion capacity. A physical examination on admission revealed an enlarged heart, a harsh systolic ejection murmur, and a decrescendo diastolic murmur in the aortic area. The electrocardiogram showed sinus rhythm with signs of left ventricular hypertrophy. The chest X-ray showed enlarged left heart chambers. Transthoracic echocardiography (TTE) revealed an ejection fraction (EF) of 43% (normal: >60%). No resting wall motion abnormality was detected, mixed aortic valve disease was confirmed, the stenosis gradient was 74/47 mmHg, the aortic valve area was 0.33 cm^2^, aortic insufficiency grade was found to be II–III, and the pressure half time value was 222 ms. Grade II tricuspid insufficiency with a pulmonary pressure of 70 mmHg was confirmed. The cardiac risk factors were hypertension, Type 2 diabetes mellitus, hyperlipidaemia, peripheral artery disease, and smoking. The right femoral common and right femoral superficial artery had been intervened prior to surgery. The left common iliac artery and left superficial femoral artery showed non-significant lesions at that time. Both common carotid arteries showed non-significant lesions. Coronary angiography revealed a non-significant proximal circumflex artery lesion. The Society of Thoracic Surgeons risk score (STS score) and EuroSCORE II before primary AVR were 0.842 and 2.8%, respectively. Aortic valve replacement was performed according to guidelines, and a LivaNova Bicarbon Slimline A-25 mm mechanical valve was implanted. Four days later, a reoperation had to be performed due to haematoma causing right heart compression, which was evacuated. Active bleeding was not revealed. Two weeks later, another reoperation had to be performed due to repeat haematoma formation in the pericardium. Discharge TTE showed good mechanical valve function with a peak gradient of 19 mmHg, and there was no sign of any PVL. Six months after discharge, the patient started to experience dyspnoea on exertion and also at rest. Upon readmission, haemolytic anaemia was discovered (Hgb: 92–82 g/dL, normal value: >130 g/dL, serum bilirubin level: 23.3 µmol/L, normal value <21 µmol/L). The patient was in a state of decompensation, partially reflected by brain natriuretic peptide level, which was 7244 pg/mL. Transoesophageal echocardiography and 3D TOE revealed severe PVL (the width of the regurgitation jet was 30–50% of the left ventricle width) located supposedly at the left coronary sinus (*Figure 1A–D*). Computed tomography angiography helped to pinpoint the largest ostium of the PVL located at the left coronary sinus and quantify the regurgitation jet (*Figure 1E–H*, Supplementary material online, *Video S1*). Computed tomography angiography discovered a chronic occlusion of the right subclavian artery, and distal filling was provided by collaterals from the right vertebral artery. The left subclavian artery was free of disease. Pleural effusion was present in both pleural cavities, which also supported the diagnosis of cardiac decompensation. Shortly after readmission, the patient became noradrenaline dependent. The calculated STS score for mortality was 5.85%, which was in line with EuroSCORE II (42.35%). Both preoperative scores represent an extremely high surgical risk for mortality. Percutaneous PVL closure represented the consensus of the heart team. Due to peripheral vascular disease in both lower extremities and right subclavian artery occlusion, a decision was made to use the left DRA as the initial approach to reduce the potential risk of vascular access site complications and bleeding risk. The left DRA was punctured with ultrasound guidance (*Figure 2A* and *B*), and a 6 French (F) Terumo radial sheath (Terumo, Japan) was introduced and later replaced with a Sheathless 8.5F MP1 catheter (Asahi, Japan; *Figure 2C*). Aortography confirmed a large-volume PVL (*Figure 3A*, Supplementary material online, *Video S2*). With fluoroscopy and TOE and 3D TOE guidance, a Gladius 0.018″ guidewire (Asahi, USA) could pass through the largest opening of the leak into the left ventricle. It was followed by a CXI 4.0 support catheter (Cook, USA). After exchanging it with Confida 0.035′ wire (Medtronic, USA), through the Sheathless guide catheter, a 14/5 mm Amplatzer Vascular plug III (AVPIII; Abbott, USA) was deployed after careful positioning to avoid mechanical valve leaflet interaction aided by TOE, 3D TOE, and fluoroscopy (*Figure 3B* and *C*). Control angiography and TOE still revealed a large-volume residual leak (*Figure 3D* and *E*, Supplementary material online, *Video S3*). With a Halberd 0.018″ wire (Asahi, Japan), it was possible to cross the left ventricle beside the prior-implanted AVPIII through the remaining leak in the ostium. A 10/5 mm AVPIII was deployed in the largest remaining leak without compromising mechanical valve function (*Figure 3F* and *G*). Final angiography revealed only a small volume of contrast entering the left ventricle through the leak (*Figure 3H*, Supplementary material online, *Video S4*). Transoesophageal echocardiography confirmed the angiography result (*Figure 4A* and *B*). By the end of the procedure, no medical haemodynamic support had to be provided (*Figure 4C*). The patient was discharged home five days later. Discharge TTE confirmed good mechanical aortic valve function, increased left ventricle EF, minimal regurgitation, and proper position of the deployed vascular plugs (*Figure 4D–F*).

**Figure 5:**
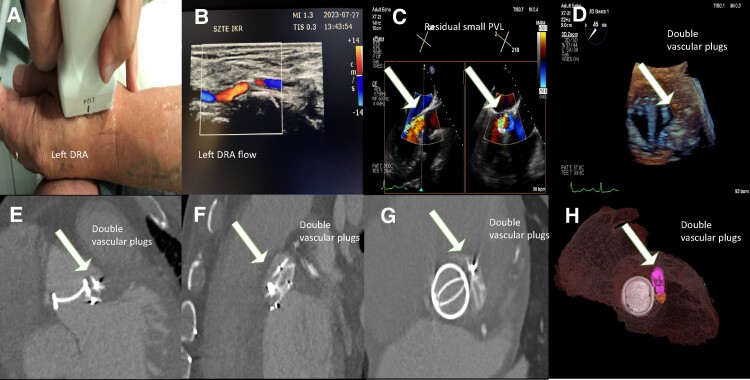
(*A*) An image demonstrating an ultrasound check of the distal radial artery 9 months after leak closure in the anatomical snuffbox. (*B*) A 9-month follow-up of the patency of the access site (the left distal radial artery) for the paravalvular leak closure procedure. (*C*) A transoesophageal echocardiography image at the 9-month follow-up after transcatheter paravalvular leak closure. The white arrows indicate a haemodynamically non-significant residual leak. (*D*) A three-dimensional transoesophageal echocardiography image at the 9-month follow-up after transcatheter paravalvular leak closure. Good mechanical valve leaflet function. A white arrow shows the treated leak position. (*E*, *F*) Computed tomography images demonstrating patent double plug positions in the prior leak location. The white arrows point at the implanted AVPIII plugs. (*G*) A computed tomography image demonstrates a patent plug position (white arrow). Good mechanical valve function. (*H*) A three-dimensional colour reconstruction of the implanted AVPIII plugs.

At a 9-month follow-up, the patency of the prior-punctured left DRA was checked by ultrasound and a patent flow with no luminal narrowing was detected (*Figure 5A* and *B*). Transthoracic echocardiography, TOE, and 3D TOE confirmed excellent vascular plug positions with only a moderate-sized residual leak (*Figure 5C* and *D*). Control CTA confirmed proper plug positions and only a small-sized residual leak was revealed. No further intervention was performed (*Figure 5E–H*, Supplementary material online, *Video S5*).

## Discussion

Previously, the gold standard for treating severe aortic mechanical valve PVL was cardiac surgery. In 1992, the first successful transcatheter aortic paravalvular closure was performed in patients.^4^ The ESC/EACTS 2021 guidelines state that reoperation due to severe PVL causing heart failure symptoms and haemolysis requiring blood transfusion is a Class I or Class IIa indication. It is also stated that transcatheter PVL closure represents a Class IIa indication and should be the treatment of choice if surgery is prohibited or represents a very high surgical risk.^5^ With the evolution of implantable devices, the number of transcatheter closures has risen, as opposed to reoperation, due to a significantly lower overall mortality risk.^5,6^ In the patient case presented here, the overall mortality risk after three prior operations was unacceptably high. By analysing TTE, 3D TOE, and CTA findings, a decision was made to perform a transcatheter closure, to place AVPIII device/s that are specifically designed for PVL closures to prevent mechanical valve leaflet interaction.^6,7^ For a procedure with large devices, at least 7F catheter size is required. If an anchor/safety wire is planned, +1F is needed.^7^ In most centres, the standard access site for aortic PVL closure is the common femoral artery.^6,8^ In the last decade, there has been a shift from femoral access toward radial access for coronary procedures even in acute settings due to decreased overall mortality, bleeding, and access site complications.^9,10^ Unlike in the case of coronary procedures, for aortic PVL closure, there are only two reports mentioning the adoption of the radial access approach.^11,12^ In the patient case presented here, the left DRA was chosen for access after careful ultrasound evaluation. Computed tomography angiography was performed, partially to evaluate arterial vascular access site options. The right subclavian artery was found to be occluded, and both the iliac and femoral arteries showed numerous lesions; thus, femoral access was avoided in an attempt to minimalize potential bleeding and access site complications. In a previous large-scale study, it has been demonstrated that DRA access is feasible for various coronary and peripheral interventions.^13^ Although ultrasound guidance is not mandatory, it is strongly recommended for DRA puncture.^14^ In their DR-BAV study, Achim *et al*.^15^ have demonstrated that with all pre-puncture ultrasound measurements, the DRA can bear an 8F or even a 9F hydrophilic sheath introducer insertion for balloon aortic valvuloplasty.

## Conclusion

Paravalvular leaks requiring further invasive treatment remain infrequent abnormalities after surgical AVR, but carry major risks for the patient. Transcatheter leak closure delivers significantly less overall risk compared with reoperation. Up to now, the radial artery access approach has not gained enough traction for performing closure procedures, even though less bleeding and less vascular complications have been documented. We have demonstrated in this report that DRA access is feasible for aortic PVL closure and carries potential advantages over even conventional radial access. However, for certain structural interventions, we should follow the evolution of access sites used in coronary interventions.

## Supplementary Material

ytae366_Supplementary_Data

## Data Availability

The data underlying this article are available in the article and in its online Supplementary material.

## Lead author biography

Dr Viktor Sasi is an interventional cardiologist who specializes in complex, mainly coronary interventions and structural interventions. He was born in Hungary. He received his medical degree in the University of Szeged, Faculty of Medicine, Hungary, in 2002. Afterwards, he specialized in internal medicine in 2007 and cardiology in 2010. Since 2005, he has been working in the Division of Invasive Cardiology in the University Clinic of Szeged, Hungary. He secured his PhD degree in 2018. He is an assistant professor in the Department of Internal Medicine, University Clinic Szeged, Hungary.

## Notes

**Consent:** The authors confirm that consent for the submission and publication of this case report, including images and associated text, was obtained from the patient in line with COPE guidance.

**Funding:** There is no funding for this report.
